# Thymosin-β4 inhibits proliferation and induces apoptosis of hepatic stellate cells through PI3K/AKT pathway

**DOI:** 10.18632/oncotarget.18748

**Published:** 2017-06-28

**Authors:** Lili Zhu, Mingliang Cheng, Yongmei Liu, Yumei Yao, Zixin Zhu, Baofang Zhang, Qiuju Mou, Yiju Cheng

**Affiliations:** ^1^ Department of Infectious Diseases, The Affiliated Hospital of Guizhou Medical University, Guiyang, Guizhou, China; ^2^ The Affliated Baiyun Hospital of Guizhou Medical University, Guiyang, Guizhou, China; ^3^ Guizhou Medical University, Guiyang, Guizhou, China

**Keywords:** Thymosin-β4, liver fibrosis, hepatic stellate cell, AKT

## Abstract

Liver fibrosis is a necessary stage for chronic liver diseases, and serious threat to human health. Hepatic fibrosis is a necessary stage for chronic liver diseases. Hepatic stellate cells (HSCs) are the primary cell type responsible for fibrosis. Thymosin beta 4 (Tβ4) has a potential role in the pathogenesis of liver fibrosis and that it is especially associated with the activation of HSCs, however, the underlying mechanisms are not fully elucidated. Herein, we investigated the potential role of Tβ4 in liver fibrosis by describing the effects of Tβ4, and we discuss the possible signaling pathway regulated by Tβ4. The expression of Tβ4 was significantly decreased in human HSC cell line LX-2 and CCl4-treated mouse liver. The depletion of Tβ4 significantly associated with the activation of HSCs via the enhanced expression of α-SMA and vimentin. Tβ4 significantly suppressed the viability and migration of HSCs. Understanding the potential effects and regulatory mechanism of Tβ4 in liver fibrosis might help to provide a novel treatment for patients with liver fibrosis.

## INTRODUCTION

Chronic liver injury triggers a wound healing response that leads to fibrogenesis and can ultimately result in the development of liver cirrhosis [1]. Activated hepatic stellate cells (HSCs) have been shown to play a function in angiogenesis and vasculogenesis. It could produce extracellular matrix (ECM) to promote the fibrogenic response [2]. The activation of resident HSCs into fibroblast-like cells is a hallmark of hepatic fibrogenesis [3].

Acitvated HSCs trigger the expression of α-smooth muscle actin (α-SMA) and production of abnormal ECM, along with enhanced proliferation and migration [4–5]. Accordingly, it becomes important to have a suitable fibrosis model for studying the underlying mechanism of HSC activation and finding therapeutic molecules for targeting liver fibrosis. It is well accepted that that Thymosin β4 (Tβ4) demonstrated anti-fibrogenic effects in HSCs [6–7]. Recently, T*β*4 has have been shown to promote the differentiation of progenitor cell lines to cardiomyocytes [8]. Several previous studies demonstrated that Tβ4 is involved in the process of fibrosis in renal fibrosis and pulmonary fibrosis. However, the underlying mechanisms of Tβ4 in liver fibrosis are not fully elucidated. In this study, our data show that Tβ4 was significantly decreased in human HSC cell line LX-2 and CCl4-treated mouse liver, and Tβ4 significantly suppressed cell viability and migration of HSCs.

## RESULTS

**Figure 1:**
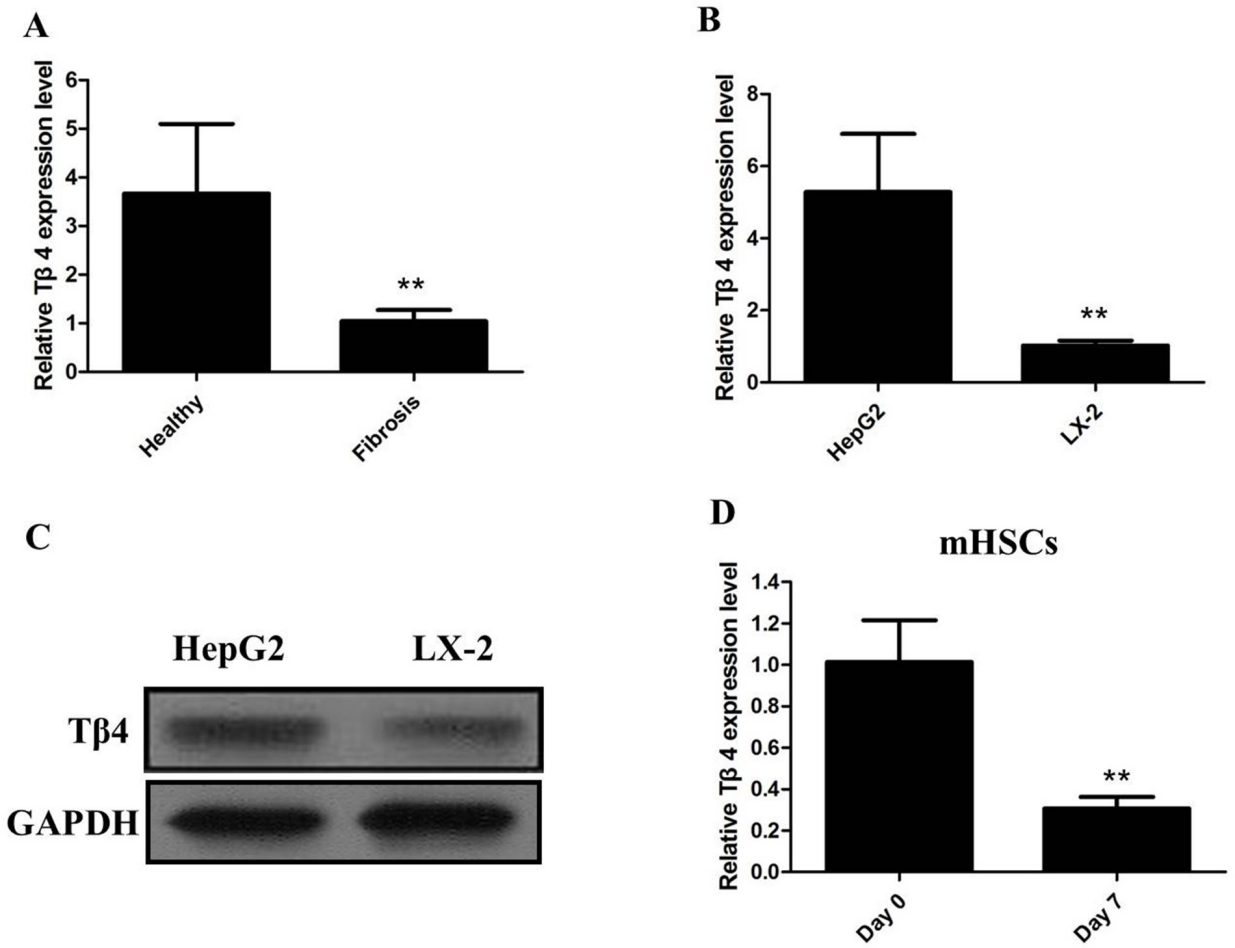
**(A)** The qRT-PCR assay revealed that the expression of Tβ4 mRNA significantly decreased in patients with liver fibrosis when compared with healthy controls (** *P* <0.01); **(B)** The qRT-PCR assay revealed that the expression of Tβ4 mRNA significantly decreased in human HSC cell line LX-2, compared with HepG2 cells (** *P* <0.01); **(C)** The western blot assay revealed that the expression of Tβ4 protein significantly decreased inhuman HSC cell line LX-2, compared with HepG2 cells; **(D)** The qRT-PCR assay revealed that the expression of Tβ4 mRNA significantly decreased in HSCs from the livers of healthy mice cultured for 7 days than freshly isolated cells (** *P* <0.01).

**Figure 2:**
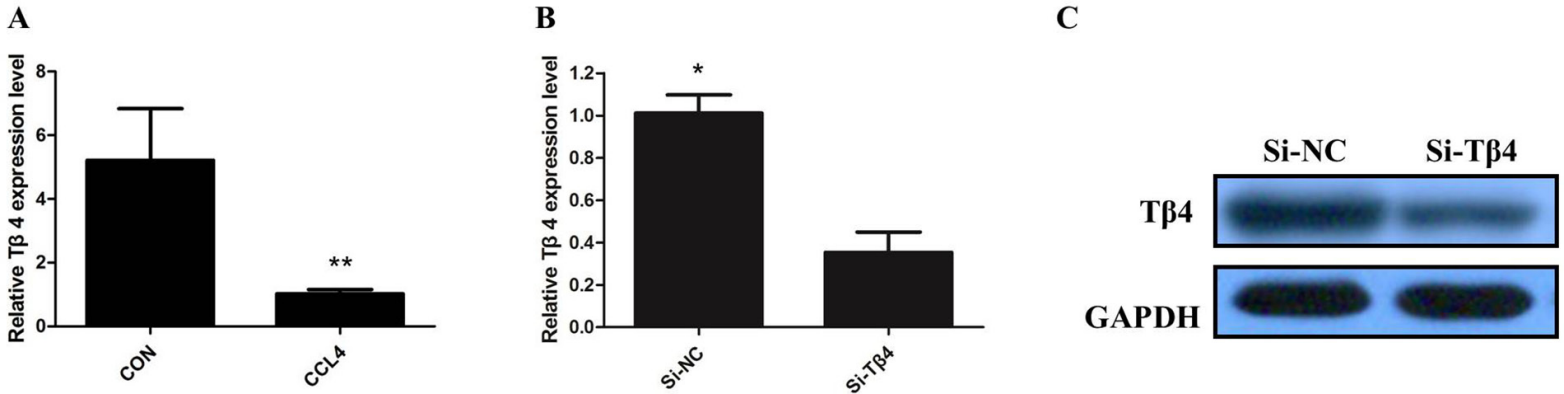
**(A)** The qRT-PCR assay revealed that the expression of Tβ4 mRNA significantly decreased in CCl4-treated liver (** *P* <0.01); **(B)** TheqRT-PCR assay revealed that Tβ4 mRNA was efficiently downexpression by transfected with siRNA in LX-2 cells (* *P* <0.05); **(C)** Thewestern blot assay revealed that the expression of Tβ4 protein was efficiently downexpression by transfected with siRNA in LX-2 cells.

We firstly detected the expression of Tβ4 mRNA in patients with liver fibrosis, our results showed that levels of Tβ4 were down-regulated in patients with liver fibrosis when compared with healthy controls (*P*<0.001; Figure 1A). The expression of Tβ4 mRNA and protein were significantly decreased in the human HSC cell line LX-2, compared with HepG2 cells (*P*<0.01, Figure 1B and 1C). Besides, the expression of Tβ4 mRNA significantly decreased in HSCs from the livers of healthy mice cultured for 7 days than freshly isolated cells (Figure 1D). Moreover, Tβ4 expression was analyzed in CCl4-induced liver fibrosis in mice. The Tβ4 expression in CCl4-treated liver was significantly lower than control liver, suggesting that Tβ4 was decreased during liver fibrosis (Figure 2A).

**Figure 3:**
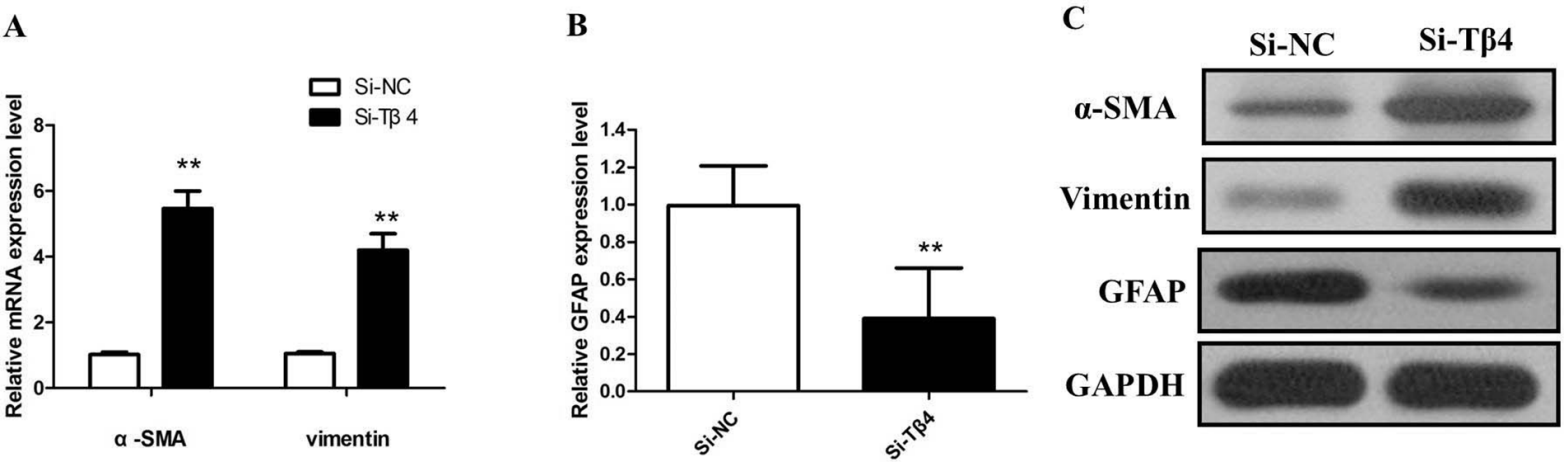
**(A)** The qRT-PCR assay revealed that mRNA levels of α-SMA and vimentin were increased in si-Tβ4 group relative to the control (** *P* <0.01); **(B)** The qRT-PCR assay revealed that mRNA levels of GFAP was decreased in si-Tβ4 group relative to the control (** *P* <0.01); **(C)** The western blot assay revealed that the expression of α-SMA, vimentin and GFAP protein in si-Tβ4 group.

Then, we knockdown the levels of Tβ4 in LX-2 cells by si-Tβ4. The qPCR and western blot assays revealed that Tβ4 expression was significantly reduced in LX-2 cell lines (Figure 2B and 2C). As expected, Tβ4 knockdown increased the expression of myofibroblastic markers including α-SMA and vimentin (*P* <0.01, Figure 3A). However, typical marker of quiescent HSC GFAP was downregulated in the Tβ4 knockdown cells (*P*<0.01, Figure 3B). Consistent with mRNA data, immunoblot analysis showed that α-SMA and vimentin protein were upregulated, and GFAP was downregulated in LX-2 cells transfected with si-Tβ4 (Figure 3C).

**Figure 4:**
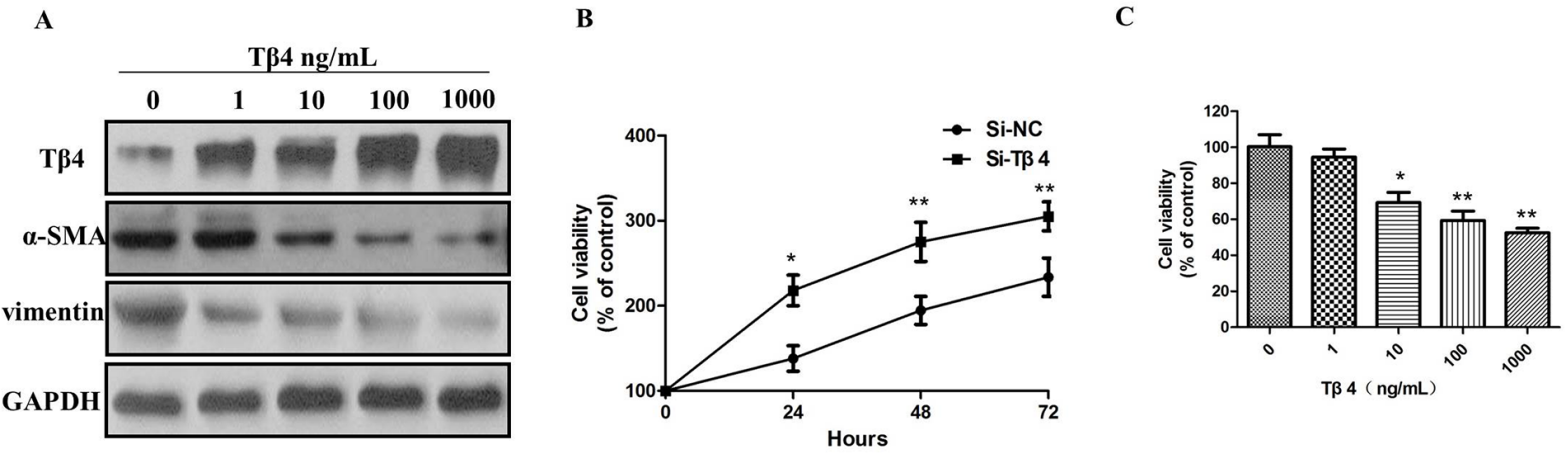
**(A)** The expression of Tβ4, α-SMA and vimentin protein in LX-2 cells treated with different concentrations of Tβ4; **(B)** CCK-8 assay Tβ4 knockdown led to a significant upregulation in cell proliferation of LX-2 cells; **(C)** The growth of LX-2 cells was significantly inhibited after treatment with different concentrations of Tβ4.

Next, LX-2 cells were treated with 1, 10, 100, 1000 ng/mL of Tβ4 and then incubated for 24 hours. The results indicated that the level of Tβ4 were upregulated after treatment with Tβ4 for 24 hours (Figure 4A). Tβ4 significantly inhibits the expression of α-SMA and vimentin, suggesting that Tβ4 can inhibit the activation of LX-2 cells by suppressing the expression of α-SMA and vimentin (Figure 4A). We then investigated the effect of Tβ4 knockdown on the proliferation of LX-2 cells by CCK-8 assays. Tβ4 knockdown led to a significant upregulation in cell proliferation of LX-2 cells (*P* <0.01, Figure 4B). Furthermore, the growth of LX-2 cells was significantly inhibited after treatment with different concentrations of Tβ4 for 24 hours (*P* <0.01, Figure 4C).

**Figure 5:**
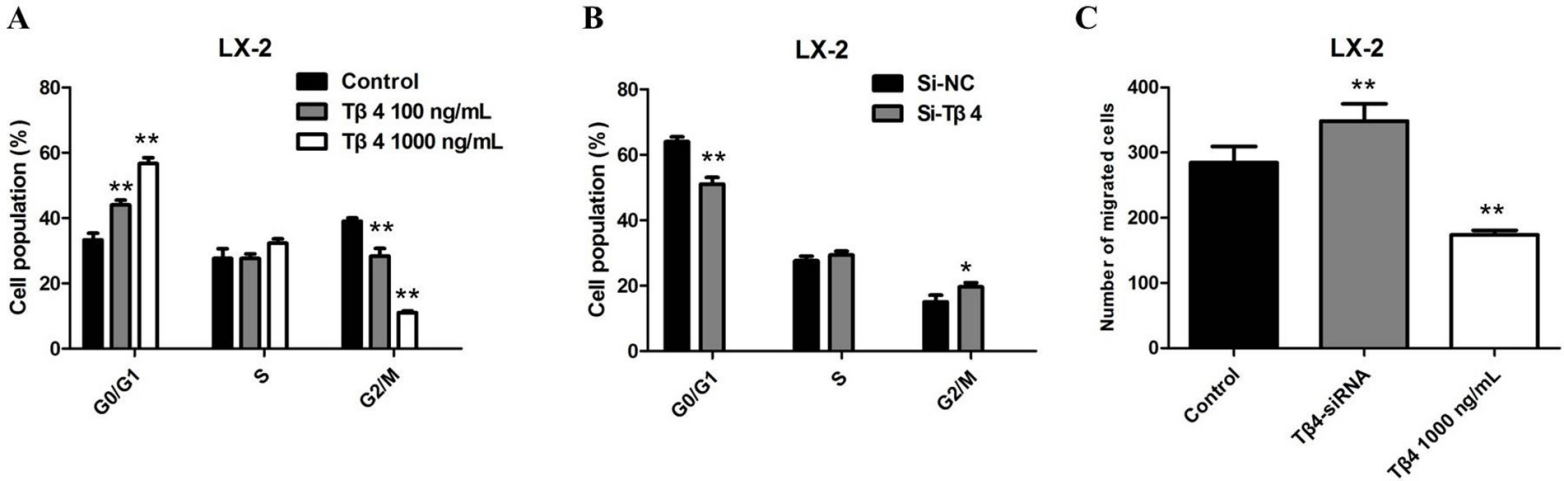
**(A)** Cell cycle analysis showed a marked increase in the number of G1 phase cells after Tβ4 treatment at a higher concentration; (B) Cell cycle analysis showed a decrease in the number of G1 phase cells after Tβ4 inhibition; (C) the results of transwell revealed that a significant increase in the number of migrated cells for LX-2 cell lines after Tβ4 depletion, whereas treating the cells with 1,000 ng/mL Tβ4 decreased the number of migrated cells compared to the control.

To further study the inhibitory effect of Tβ4 on cell growth, we investigated the cell cycle distribution by flow cytometry. Cell cycle analysis showed that Tβ4 treatment lead to a marked increase in the the number of G1 phase cells, however, suppression of Tβ4 decreased the G0/G1 phase percentage of LX-2 cells, suggesting that inhibition of Tβ4 expression may interfere with cell cycle progression (*P* <0.01, Figure 5A–5B). In addition, we utilized the Transwell analysis to examine the effects of Tβ4 on the migration of LX-2 cells. The results revealed that Tβ4 inhibition significantly promoted the migration capability of LX-2 cells, whereas a significant decrease in the number of migrated cells for LX-2 cell lines after treatment of 1000 ng/mL Tβ4 (Figure 5C).

**Figure 6:**
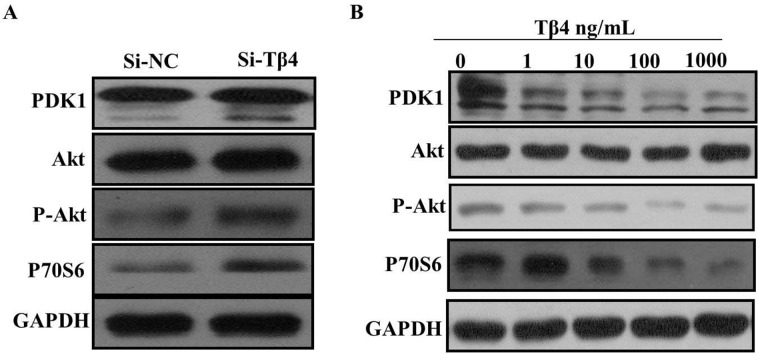
**(A)** knockdown of Tβ4 expression upregulated the expression of p-Akt, PDK1 and P70S6 in LX-2 cells; **(B)** Tβ4 treatment significantly inhibited the expression of p-Akt, PDK1 and P70S6.

Because PI3K/Akt signaling pathway is vital for liver fibrosis, we next examined whether Tβ4 could regulate the expression of p-Akt, PDK1 and P70S6. As expected, the expression of p-Akt, PDK1 and P70S6 can be induced by Tβ4 knockdown (Figure 6A). Correspondingly, Tβ4 treatment significantly inhibited the p-Akt, PDK1 and P70S6 (Figure 6B).

## DISCUSSION

Liver fibrosis is a progressive pathological process as part of the wound healing and tissue remodeling mechanism in response to chronic liver insults [9]. Activation of HSCs have shown the critical role on liver fibrosis. During liver fibrogenesis, ECM is produced and accumulated, leading to liver dysfunction and irreversible cirrhosis [10]. It is known that the principle cellular source of ECM are HSCs. Thus, suppression of HSC activation may be a potential targeted therapy for liver fibrosis.

Tβ4 is involved in many critical biological processes including fibrosis, collagen deposition, and blood vessel [11]. It has been confirmed that Tβ4 could act as anti-inflammatory and anti-fibrotic agents *in vitro* and *in vivo* [12–13]. However, the exact role and function of Tβ4 in liver fibrosis remains unclear. In the current study we utilized mouse and cell models to investigate the effects of Tβ4 on liver fibrosis *in vitro* and *in vivo*. Here we demonstrated that the Tβ4 levels were significantly downregulated in HSC cell line LX-2. In addition, Tβ4 was decreased in human liver with advanced liver fibrosis.

Tβ4 exerted an anti-fibrotic effect in the acute liver injury model [14]. Our study comfirmed that Tβ4 could supress liver fibrosis by inhibiting the proliferation and migration of HSCs. In this study, our comprehensive analyses found that Tβ4 was related to HSC activation. To evaluate the functions of Tβ4 in the activated HSCs, LX-2 cells were transfected with a Tβ4-specific siRNA. Our subsequent studies showed that inhibition of Tβ4 could upregulate the α-SMA and vimentin expression, however, treatment with Tβ4 significantly inhibits the expression of α-SMA and vimentin in LX-2 cells, suggesting that Tβ4 can inhibit the activation of HSCs.

Tβ4 is a candidate regulator of cell proliferation and migration with important roles in distinct protrusion-related processes. Tβ4 is believed to have an anti-migratory function because it could induce cytoskeletal disorganization and inhibit actin polymerization. In our study, knockdown of Tβ4 increased cell proliferation of LX-2 cells. The result of inhibition of growth was confirmed by treatment with different concentrations of Tβ4 for 24 hours in LX-2 cells. Moverover, inhibition of Tβ4 induces LX-2 proliferation and migration by regulating the PI3K/AKT pathway. The PI3K/AKT pathway is one of targets of Tβ4. Our western blot assay showed that knockdown Tβ4 increased the expression of p-Akt, PDK1 and P70S6, however, Tβ4 treatment significantly inhibited the expression of p-Akt, PDK1 and P70S6.

Our current study demonstrates that Tβ4 is decreased in the activated HSCs. Exogenous Tβ4 reduces liver fibrosis by inhibiting the proliferation and migration of HSCs. Tβ4 represents a potential therapeutic target for liver fibrosis.

### Statistical analysis

Data from at least three independent experiments were expressed as the mean ± SD. Differences between multiple groups were evaluated using one-way analysis of variance. Differences between two groups were compared using a Student's *t*-test. *P* < 0.05 was considered significant. All statistical analyses were performed with SPSS software (version 13; SPSS, Chicago, IL).

## MATERIALS AND METHODS

### Cell lines and human samples

The human hepatic stellate cell line LX-2 and HepG2 derived from the human hepatocellular carcinoma (HCC) were cultured in DMEM supplemented with 10% fetal bovine serum and 1% penicillin-streptomycin at 37°C in 5% CO_2_. Human fibrotic liver tissues (n = 22) and healthy liver tissues (n = 20) were obtained from patients who had underwent surgery at The Affiliated Hospital of Guizhou Medical University. Primary HSCs were isolated from normal C57BL/6 mice. This study was approved by the ethics committee of Guizhou Medical University.

### CCl4 liver injury model

Eight-week-old male C57BL/6J mice (*n* = 6) received intraperitoneal injection of 7 μL/g of 10% CCl4 (Sigma-Aldrich, St. Louis, MO, USA, cat# 289116) in olive oil two times weekly for six weeks. Meanwhile, mice (*n* = 6) treated with olive oil treatment were considered as the control mice. The animals were provided by the Experimental Animal Center of Guizhou Medical University. The animal experimental protocol was approved by the University Animal Care and Use Committee of Guizhou Medical University. Mice were sacrificed under anesthesia at the end of six weeks and the livers were removed for further analysis. The liver tissues were used for Masson staining by fixation with 10% formalin. Quantitative analysis for the Masson-positive area was calculated from five fields for each liver slice.

### RNA isolation and quantitative RT-PCR

Total RNA was extracted from cells using Trizol (Invitrogen, Carlsbad, CA, USA) according to the manufacturer's instructions. After assuring RNA quality and concentration, total RNAwas used to synthesize cDNA using the SuperScript II First-strand Synthesis System (Invitrogen) following the manufacturer's instructions. Gene expression was evaluated by qRT-PCR analysis. The mRNAs were quantified by realtime RT-PCR using Power SYBR Green Master Mix (Applied Biosystem) according to the manufacturer's specifications (Eppendorf Mastercycler, Real-Time PCR). Samples were analyzed in triplicate according to the Delta-Delta threshold (ΔΔCt) method. GAPDH was used as an internal control. The following primer sequences were used for qRT-PCR. Human Tβ4 primers, forward: CGC AGA CCA GAC TTC GCT CGT AC; reverse: TCC TTC CCT GCC AGC CAG ATA GAT; Human α-SMA primers, forward: AAT GGC TCT GGG CTC TGT AA; reverse: CTT TTC CAT GTC CCA GT; Human vimentin primers, forward: CGA AAA CAC CCT GCA ATC TT; reverse: GTG AGG TCA GGC TTG GAA AC; Human GFAP primers, forward: CTG GAG GTT GAG AGG GAC AA; reverse; CAG CCT CAG GTT GGT TTC AT; GAPDH, forward: GGG AGC CAA AAG GGT CA; reverse: GAG TCC TTC CAC GAT ACC AA-3.

### Western blot analysis

Protein extracts were run on SDS acrylamide gels and transferred onto nitrocellulose. Blots were incubated with anti-Tβ4, PDK1, Akt, phospho-Akt (Ser473), P70S6, α-SMA, Vimentin, GFAP, and GAPDH (Cell Signaling Technology, Beverly, MA, USA) overnight at 4°C. After incubation with secondary horseradish-peroxidase conjugated antibodies (Santa Cruz Biotechnology), the bands were visualized by the enhanced chemiluminescence light method (Amersham Biosciences) and exposed to X-omat film (Eastman Kodak Co., New Haven, CT) or a chemiluminescence imager (Image Station 2000R, Eastman Kodak Co.).

### Tβ4 siRNA transfection

Silencing of the Tβ4 gene was achieved by transfecting cells with small interfering RNA (siRNA). The pooled Tβ4 siRNA duplexes were synthesized by Shanghai Genepharma Co., Ltd. Cells were transfected with Tβ4 siRNAs (30 nM; 5’-UCGAUAAGUCGAAACUGAATT-3’) for 24 hours using Lipofectamine 2000 (Invitrogen, Carlsbad, CA, USA) according to the manufacturer's instructions. Cells were transfected with negative control siRNA using the same protocol. Cells were harvested and total RNA was extracted for gene expression analysis. The efficiency of gene knock down was evaluated by qRT-PCR and Western Blot analysis.

### Cell viability

Cells were cultured to the logarithmic phase and then inoculated onto 96-well plates (10^4^ cells/well) for 24 h. Cells were cultured for 24 h after treatment, cell proliferation was detected using the Cell Counting Kit-8 (CCK-8) reagent kit (Dojindo Molecular Technologies, Inc., Shanghai, China).

### Detection of cell cycle

Cells were inoculated onto a 6-well plate at 5 × 10^5^ cells/well. After treatment of Tβ4 (Gift by RegeneRx company) for 48 h, the cells were collected and washed twice with pre-cooled PBS. Cells were fixed in pre-cooled 75% ethanol in a 4°C refrigerator overnight, washed with PBS twice, and stained with propidium iodide (PI) containing RNase in the dark for 30 min. Cell cycle phase was detected using a flow cytometer (FACS420, BD Biosciences, San Jose, CA).

### Transwell migration assay

Cells were placed in the top chamber of transwell migration chambers (8 μm; Millipore, Billerica, MA, USA). After 48 h, cells which had not migrated to the lower chamber were removed from the upper surface of the transwell membrane with a cotton swab. Migrating cells on the lower membrane surface were fixed, stained, photographed and counted using a microscope at ×100. Experiments were assayed in triplicate, and ≥5 fields were counted in each experiment.
